# From pomiculture waste to biotechnological raw material: efficient transformation using ligninosomes and cellulosomes from *Pleurotus* spp.

**DOI:** 10.1186/s40643-022-00555-x

**Published:** 2022-06-13

**Authors:** Jasmina Ćilerdžić, Milica Galić, Mirjana Stajić

**Affiliations:** Faculty of Biology, University of Belgrade, Takovska 43, 11000 Belgrade, Serbia

**Keywords:** Cellulolytic enzymes, Ligninolytic enzymes, *Pleurotus* spp., Pomiculture residues

## Abstract

The goal of this study was to determine the capacity of *Pleurotus* spp. lignocellulosome to transform frequent pomiculture residues (grapevine-, plum-, and raspberry sawdust) into raw materials for biotechnological processes. All three lignocellulosics induced the synthesis of ligninolytic and cellulolytic enzymes in the tested species. Laccase was dominant in the ligninolytic cocktail, with a maximum activity of 40,494.88 U L^−1^ observed after the cultivation of *P. pulmonarius* on grapevine sawdust. Grapevine sawdust also proved to be the optimal substrate for the synthesis of versatile peroxidases especially in *P. eryngii* (1010.10 U L^−1^), while raspberry sawdust favored the production of Mn-dependent peroxidase in *P. pulmonarius* (479.17 U L^−1^). *P. pulmonarius* was the dominant cellulolytic agent and raspberry sawdust was optimal for the synthesis of xylanases, and endo- and exo-cellulases (15,746.35 U L^−1^, 9741.56 U L^−1^, and 836.62 U L^−1^), while grapevine sawdust mostly induced β-glucosidase activity (166.11 U L^−1^). The degree of residues delignification was more substrate- than species-dependent, ranging between 6.44 and 23.72% after the fermentation of grapevine and raspberry sawdust with *P. pulmonarius*. On the other hand, the lowest level of cellulose consumption was also observed on raspberry sawdust after the cultivation of *P. eryngii*, which together with high delignification also induced the highest selectivity index (1.27). The obtained results show the exceptional lignocellulolytic potential of *Pleurotus* spp. enzyme cocktails which opens up many possibilities for their application in numerous biotechnological processes.

**Graphical Abstract:**

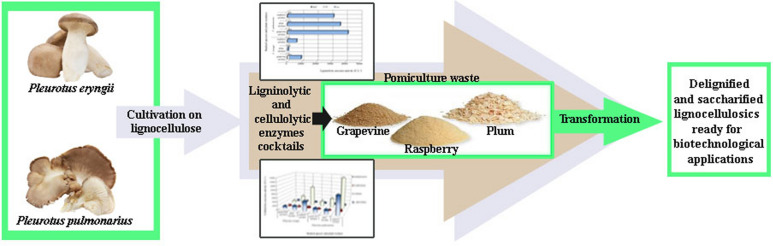

## Introduction

Despite the high percentage of holocellulose (cellulose and hemicellulose) in various agricultural lignocellulosic residues, its utilization is limited by binding tightly to lignin which protects it from hydrolytic enzymes attack (Hammel 1997; Prasad et al. 2019). Therefore, the most important step in the effective conversion of lignocellulose into value-added products is its delignification, i.e., the release of holocellulose from the lignin protective shield making it available for saccharification and further industrial purposes (Sánchez 2009; Bimestre et al. 2022). This is the most demanding part of the lignocellulose pretreatment process, limited by numerous environmental and economic shortcomings. Namely, conventional physicochemical methods for removing lignin from lignocellulose feedstocks imply the use and formation of numerous harmful chemical compounds, some of which can inhibit the process itself, and also involve high energy consumption (Saha et al. 2016). An alternative method is the biological delignification of lignocellulose, which is both safe for the environment and less expensive. Despite the obvious advantages of biological over conventional treatments, they also involve certain disadvantages, such as being more time-consuming and less efficient. Therefore, one of the main current scientific tasks is to find the most efficient biodegradable system as well as the conditions for its maximum expression. It is well known that the most promising candidates for the biological pretreatment of lignocellulose are white-rot fungi and their ligninosomes composed mainly of peroxidases and laccases (Leonowicz et al. 1999; Ghaffar et al. 2015; Fang et al. 2018). They differ greatly in terms of the pattern of lignocellulose depolymerization, i.e., whether they simultaneously degrade lignocellulose polymers or selectively degrade lignin (Saha et al. 2016). Numerous studies have shown that the efficiency and selectivity of delignification are greatly affected not only by the genetic potential of the species, but also by its affinity for lignocellulosic substrates differing in chemical composition. Thus, species of the genera *Phanerochaete*, *Pleurotus*, *Ganoderma*, and *Trametes,* etc., have already been confirmed as successful delignificators of oil palm residues, rice straw, oak sawdust, and wheat straw, respectively (Knežević et al. 2014; Piñeros-Castro and Velásquez-Lozano 2014; Ćilerdžić et al. 2016a; Mustafa et al. 2016). The microbial enzymatic hydrolysis of delignified substrates is also an important step in the economical and sustainable transformation of plant waste, which depends on numerous factors, including the selection of the species/strain and substrate type (Yoon et al. 2014; Bhardwaj et al. 2021). The most commonly used species for the commercial production of cellulases are micromycetes from the genera *Aspergillus* and *Trichoderma*, although some macromycetes belonging to the genera *Phanerochaete*, *Schizophyllum*, and *Pleurotus* also showed significant cellulolytic potential (Lynd et al. 2002; Goyal and Sony 2011).

Numerous lignocellulosic wastes, available locally or globally (from rice straw and sugarcane bagasse in southeast Asia to wheat straw and corn stalks available worldwide) have been studied for their suitability to induce ligninolytic enzymes synthesis in white-rot fungi during fermentation (Bilal et al. 2017). However, despite the proven potential for the induction of ligninolysis and their use in biotechnological processes and industry, the main disadvantage of substrates such as straw and reed is the competitiveness for use as animal feed. On the other hand, numerous lignocellulosic residues have not been explored yet, despite their considerable abundance and great disposal problems. Thus, only a few studies have dealt with the fermentation of pomiculture lignocellulosic residues, such as cherry sawdust, mandarin peels, and grapevine sawdust, and their potential to induce the synthesis of ligninolytic enzymes in several white-rot species (Stajić et al. 2006, 2017; Simonić et al. 2010; Ćilerdžić et al. 2018).

Starting from the great nutritional and medicinal potential of *Pleurotus eryngii* (DC.:Fr.) Quél. and *P. pulmonarius* (Fr.) Quél., which are increasingly produced commercially worldwide, our study aimed at determining their potential to depolymerize abundant but poorly studied pomiculture residues which could be their cultivation substrates. The main goals of the study were the characterization of ligninolytic and cellulolytic enzymes synthesized by *P. eryngii* and *P. pulmonarius* during the fermentation of grapevine-, plum- and raspberry sawdust as well as defining their depolymerization selectivity.

## Materials and methods

### Organism and growth conditions

*Pleurotus eryngii* HAI 1017 and *P*. *pulmonarius* HAI 509 cultures obtained from the Institute of Evolution, University of Haifa, Israel (HAI), are maintained on Malt agar medium at 4 °C in the culture collection of the Institute of Botany, Faculty of Biology, University of Belgrade.

The inoculum preparation was performed by inoculating 100.0 mL of the synthetic medium (glucose, 10.0 g L^−1^; NH_4_NO_3_, 2.0 g L^−1^; K_2_HPO_4_, 1.0 g L^−1^; NaH_2_PO_4_ x H_2_O, 0.4 g L^−1^; MgSO_4_ × 7H_2_O, 0.5 g L^−1^; yeast extract, 2.0 g L^−1^; pH 6.5) with 25 mycelial disks (Ø 0.5 cm) of a 7-day culture. The incubation was performed on a rotary shaker (22 ± 2 °C, 160 rpm) for 7 days. The obtained biomass was washed three times using sterile distilled water (dH_2_O) and then homogenized with 100.0 mL of dH_2_O in a laboratory blender (Waring, USA). Solid-state cultivation was carried out at 25 °C in the dark, using 250-mL flasks containing 6.0 g of pomiculture residue (grapevine-, plum-, and raspberry sawdust) as the only carbon source and the addition of 30.0 mL of the synthetic medium without glucose. Thus prepared and sterilized (114 °C, 15 min) substrates were inoculated with 9.0 mL of homogenized inoculum.

### Enzyme assays

The extracellular enzymes were extracted after 21 days of cultivation by stirring the samples with 50.0 mL dH_2_O on a magnetic stirrer (4 °C, 10 min). The extracts were centrifuged (at 4 °C and 3000 rpm, for 15 min), and the obtained supernatants were used for the spectrophotometric (BioQuest CECIL CE2501, UK) determination of the activities of Mn-oxidizing peroxidases, laccases, exo-cellulases, endo-cellulases, β-glucosidases, and xylanases. The activities of the Mn-oxidizing peroxidases [Mn-dependent peroxidases (MnP, EC 1.11.1.13), versatile peroxidase (VP, EC 1.11.1.16)] and laccases (EC 1.10.3.2) were determined using 3 mM phenol red (ε_610_ = 22 000 M^−1^ cm^−1^) and 2,2'-azino-bis-[3-ethyltiazoline-6-sulfonate] (ABTS; ε_436_ = 29,300 M^−1^ cm^−1^), respectively, as the substrates (Stajić et al. 2010). The enzymatic activity of 1 U was defined as the amount of enzyme which transforms 1 μmol of substrate per minute.

Microcrystalline cellulose (1%) and medium viscosity carboxymethyl cellulose (1%) were used as the substrates for the determination of exo- and endo-cellulase activity, respectively, with glucose as the standard. Dinitrosalicylic acid (DNS) reagent was utilized to stop the reaction and the fermentation efficiency, i.e., the concentration of the reducing sugars, was measured spectrophotometrically at 540 nm (Bernfeld 1955). One unit of exo- and endo-cellulase activity was defined as the amount of enzyme required to produce 1.0 µmol of glucose per minute at 39 °C. The activity of xylanase was estimated using birchwood xylan (1%) as the substrate and xylose as the standard, while the DNS reaction was also performed for the detection of released xylose. The unit of xylanase activity was defined as the amount of enzyme required to produce 1.0 µmol of xylose per minute at 39 °C. 4-Nitrophenyl β-d-glucopyranoside and p-nitrophenol were used as the substrate and standard, respectively, for the determination of β-glucosidase activity, and 1 M Na_2_CO_3_ was used as the stopping reagent prior to the measurement of absorbance at 405 nm (Grujić et al. 2015). One unit of β-glucosidase activity was defined as the amount of enzyme required to produce 1.0 µmol of p-nitrophenol per minute at 37 °C.

Specific enzymes activities (U mg^−1^) were obtained as the ratio between absolute enzymes activities (U L^−1^) and total proteins content (mg mL^−1^) determined by Bradford's method, using bovine serum albumin as the standard (Silva et al. 2005).

### Electrophoresis

The isoforms of the most active enzyme were screened for both *Pleurotus* species after fermentation of all the tested pomiculture residues. Laccase isoforms and their isoelectric points (pIs) were detected by isoelectric focusing (IEF) in a 7.5% polyacrylamide gel with 5% ampholyte in the 3–10 pH range using a Mini IEF cell 111 (Bio-Rad, USA). The zymogram was visualized after the incubation of the gel in a mixture composed of 10 mM ABTS and 200 mM phosphate buffer (pH 5.0) at room temperature. After focusing completion, trichloroacetic acid was used for gel fixation, while Coomassie Brilliant Blue was utilized for staining the protein bands. An IEF marker in the pI range from 3.6 to 9.3 (Sigma-Aldrich, USA) was used.

### Determination of fibers content

The modified methods of Kirk and Obst (1988) and Van Soest et al. (1991) were used for the determination of the hemicellulose, cellulose, and lignin contents. Initially, a solution of neutral detergent and Na_2_SO_3_ was used for the treatment of the dried ground samples under reflux conditions to remove the soluble sugars, proteins, lipids, and vitamins, and the obtained biomass presented neutral detergent fibers (NDFs). Hemicellulose was then removed with a solution of acidic detergent and the content of acidic detergent fibers (ADFs) was measured. The difference in mass between the NDFs and ADFs represented the hemicellulose content in the samples. ADFs were further used for the determination of the lignin content (LC) by the incubation of the samples in 72% H_2_SO_4_ at 30 °C and hydrolysis at 120 °C. The LC was expressed as the percentage present in the original sample. Finally, the cellulose content was calculated as the difference between the ADFs and LC.

### Statistical analyses

All the experiments were done in three replicates and the results were expressed as mean ± standard error. One-way analysis of variance (ANOVA) and Tukey's test were performed using STATISTICA, version 6.0 (StatSoft, Inc., Tulsa, USA) to test any significant differences between the means. Statistical significance was declared at *p* < 0.05.

## Results

### Ligninolytic enzymes

**Fig. 1:**
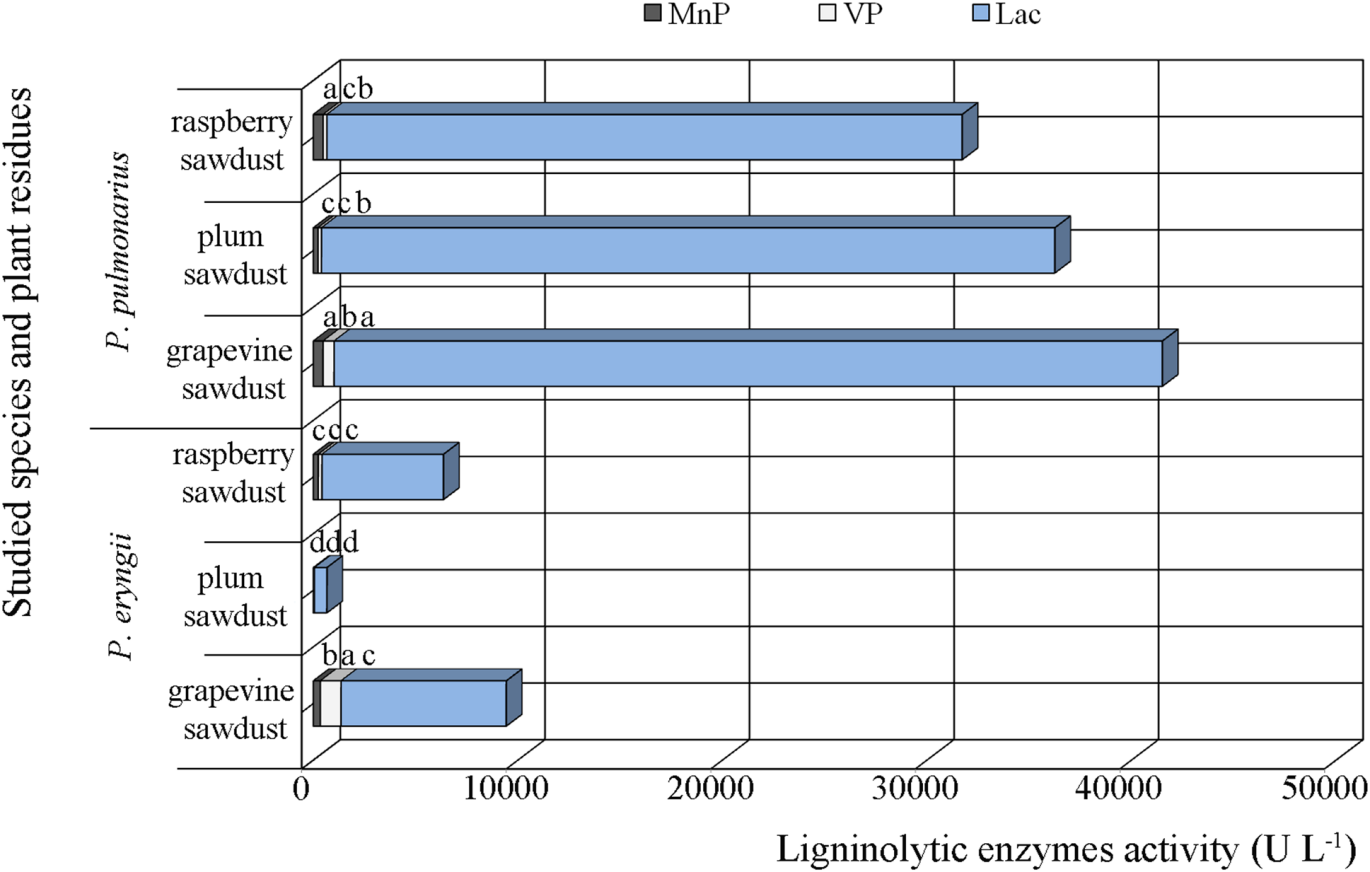
Activity of Mn-dependent peroxidases, versatile peroxidases and laccases of *Pleurotus* spp. depending on the type of pomiculture residues. The values with the same superscript letter (for each enzyme) are not significantly different (*p* < 0.05)

*Pleurotus eryngii* and *P*. *pulmonarius* produced all three ligninolytic enzymes during their cultivation on the tested pomiculture residues (Fig. 1). However, laccase was the dominant synthesized enzyme on all three lignocellulosics fermented by both studied species, but with several times higher activity obtained in *P*. *pulmonarius*. Thus, the maximal laccase activity (40,494.88 ± 119.45 U L^−1^) was measured after cultivation on grapevine sawdust, whereas slightly lower values were observed on plum and raspberry sawdusts. Approximately, fivefold lower levels of laccase activity was obtained on grapevine and raspberry sawdusts fermented by *P*. *eryngii* (8077.36 and 5938.57 U L^−1^, respectively), while plum sawdust did not favor the synthesis of this enzyme as only 606.29 U L^−1^ was measured. Plum sawdust also proved to be an unfavorable substrate for the induction of Mn-dependent peroxidase and versatile peroxidase activity, especially in *P. eryngii*, while grapevine sawdust was again optimal as in the case of laccases. The maximum level of MnP activity was measured in *P*. *pulmonarius* after raspberry sawdust fermentation (479.17 ± 9.47 U L^−1^), while a slightly lower value was obtained on grapevine sawdust fermented with the same species (473.48 ± 7.58 U L^−1^). Grapevine sawdust also favored the synthesis of MnP in *P. eryngii* reaching an activity level of 344.70 U L^−1^, while for plum sawdust the activity level of this enzyme was tenfold lower (Fig. 1). Grapevine was particularly favorable for the VP production in both *Pleurotus* species, but the absolute maximum of 1010.10 U L^−1^ was measured in *P. eryngii*, while the value (540.40 ± 44.30 U L^−1^) obtained for *P. pulmonarius* was twice as low. Significantly lower VP activity of approximately 180.00 U L^−1^ was measured on the other two lignocellulosic substrates with the exception of plum sawdust fermented with *P. eryngii* when a minimum of only 25.85 U L^−1^ was obtained (Fig. 1).

**Fig. 2:**
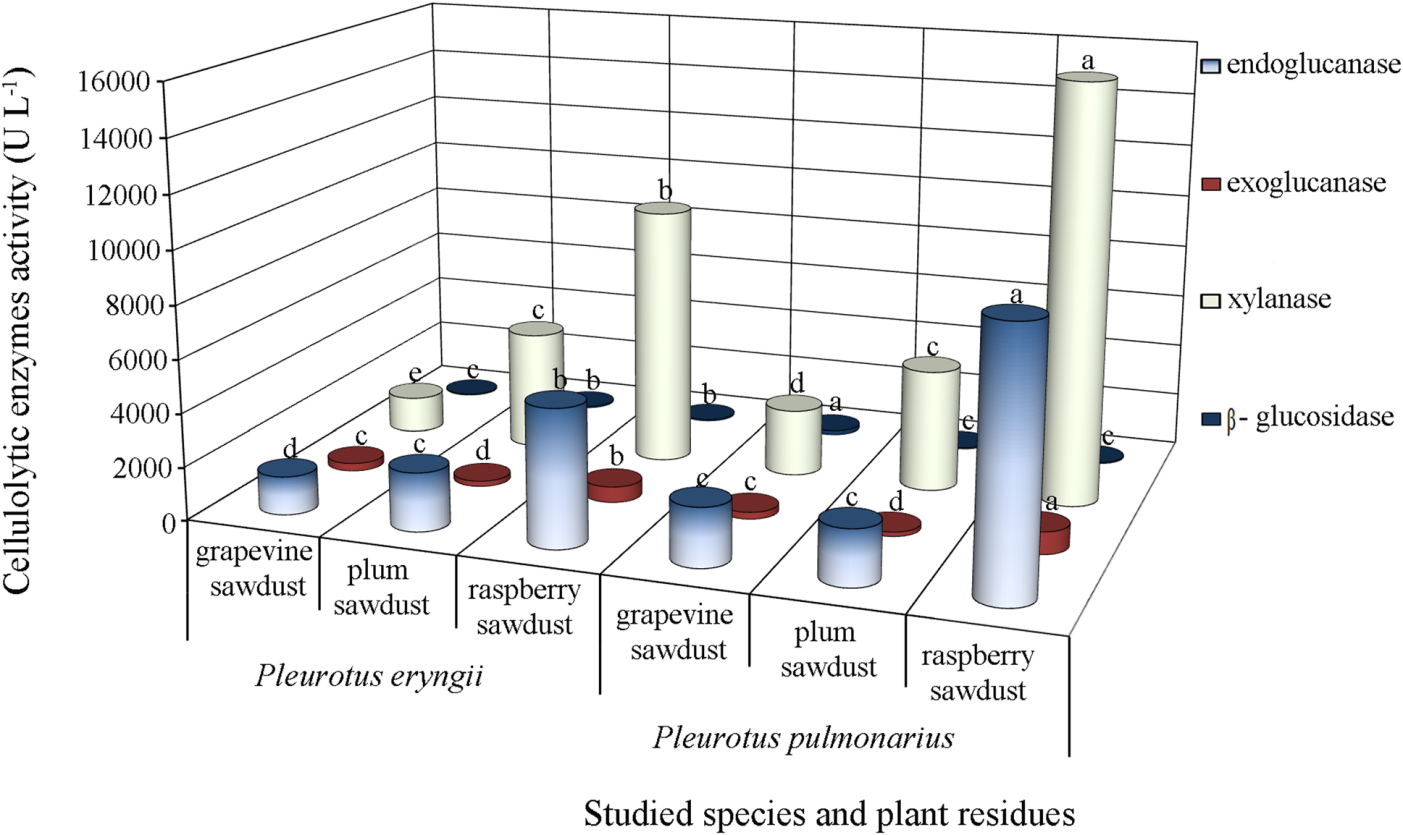
Isoelectric focusing pattern of *Pleurotus eryngii* (1) and *P*. *pulmonarius* (2) laccases after the fermentation of pomiculture residues

Due to the highest activity, laccases were chosen for further detailed profiling. The obtained laccase zymogram not only varied among the studied species, but also differed for each species on various substrates (Fig. 2). Thus, the number, intensity, and pI of the visualized laccase isoforms obtained for the species varied on different substrates. However, it should be emphasized that the number and intensity of isoforms positively correlated with the measured activity as the highest number of isoforms was visualized on grapevine sawdust formerly shown as the best inducer of activity in both the studied species. Hence, several isoenzymes with pIs in the range of 3.6 and 4.6 were synthesized by both *P*. *eryngii* and *P*. *pulmonarius*, while one less intense isoform with a pI of 5.1 was visualized only for *P*. *eryngii* (Fig. 2). Both of the tested species also produced isoenzymes with a pI of about 3.6 on plum and raspberry sawdusts which were visualized as strong bands, while differing in bands with higher pIs. Thus, isoenzymes with a pI of about 5.1 were active in both the tested species during the fermentation of plum sawdust, inducing one more isoform in *P*. *pulmonarius* with a pI of about 4.6. The highest diversity between the tested species in terms of secreted isoforms was obtained on raspberry sawdust as only one isoform was synthesized by *P*. *Pulmonarius,* while several bands of pIs between 3.6 and 5.1 were visualized for *P*. *eryngii* (Fig. 2).

### Cellulolytic enzymes

**Fig. 3:**
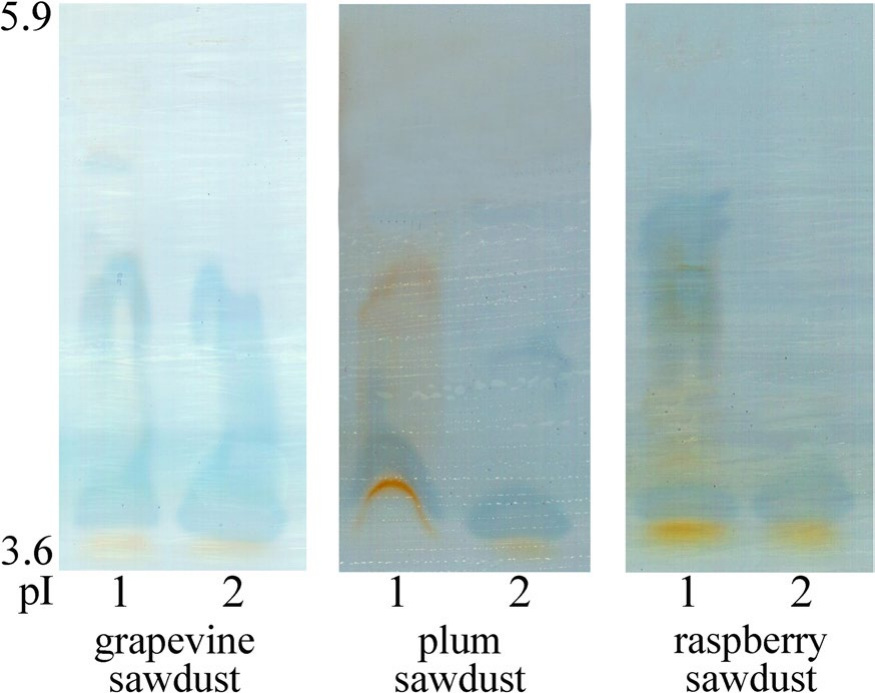
Activity of endo-glucanases, exo-glucanases, xylanases and β-glucosidases of *Pleurotus* spp. depending on the type of pomiculture residues. The values with the same superscript letter (for each enzyme) are not significantly different (*p* < 0.05)

All the cellulolytic enzymes examined in the study were produced by the tested *Pleurotus* species on all pomiculture wastes (Fig. 3). However, *P*. *pulmonarius* convincingly dominated in the synthesis of all four enzymes compared to *P*. *eryngii*. Raspberry sawdust proved to be the best substrate for endo- and exo-glucanase as well as xylanase synthesis, while grapevine sawdust was the most favorable for β-glucosidase production. Xylanase was the most dominant enzyme with activity peaks as high as 15,746.35 U L^−1^ in *P*. *pulmonarius* and 9771.53 U L^−1^ in *P*. *eryngii* after cultivation on raspberry sawdust. However, the activity of this enzyme was significantly lower on two other lignocellulosic substrates with a minimum of 1371.65 U L^−1^ obtained on grapevine sawdust fermented by *P*. *eryngii* (Fig. 3). Raspberry sawdust induced the synthesis of highly active endo-glucanase isoforms, especially in *P*. *pulmonarius*, with the value of 9741.56 U L^−1^, which was almost twice as high as in *P*. *eryngii*. Significantly lower endo-glucanase activity was measured after the fermentation of grapevine and plum sawdust by both species, ranging from 1447.16 to 2216.33 U L^−1^. The exo-glucanase activity synthesized by both *Pleurotus* species on all the studied substrates was many-fold lower than endo-glucanase and xylanase, but the same substrate enhanced its activity maximally. Thus, the peaks of exo-glucanase activity in *P*. *pulmonarius* (836.62 ± 49.95 U L^−1^) and *P*. *eryngii* (597.17 ± 52.18 U L^−1^) were achieved after raspberry sawdust fermentation, while plum sawdust was the least favorable for its synthesis (165.37 U L^−1^ and 202.29 U L^−1^, respectively). Extremely low activity of β-glucosidase was observed for both the studied species on the tested lignocellulose residues, ranging from 44.47 U L^−1^ synthesized by *P*. *eryngii* on grapevine sawdust to 166.11 U L^−1^ produced by *P*. *pulmonarius* on the same substrate (Fig. 3).

### Depolymerization of lignocellulosic residues

The total dry matter loss of the tested pomiculture residues after the cultivation of *Pleurotus eryngii* and *P. pulmonarius* was species- and substrate-dependent (Table 1). It ranged from 10.63% in grapevine sawdust fermented by *P*. *eryngii* to 22.17% in raspberry sawdust fermented by *P*. *pulmonarius*. The tested species differed significantly in the induction of dry matter loss on the grapevine- and raspberry sawdusts, in contrast to plum sawdust, where a uniform dry matter loss of approximately 12% was observed. The extent of delignification of the tested pomiculture residues was rather substrate- than species-dependent, varying between 6.44 and 23.72% in the grapevine and raspberry sawdusts, respectively, after fermentation by *P*. *pulmonarius* (Table 1). Slightly lower delignification values were also recorded after fermentation of raspberry sawdust with *P*. *eryngii* (19.82%) and plum sawdust with *P*. *pulmonarius* (19.67%). A significantly lower lignin consumption was observed following the cultivation of both the studied species on grapevine sawdust. The highest percentage of holocellulose was removed from the plum sawdust by *P*. *pulmonarius* which consumed 24.89% of cellulose and 59.44% of hemicellulose (Table 1). On the other hand, the lowest level of cellulose degradation was obtained after the cultivation of *P*. *eryngii* on raspberry sawdust which, together with a high percentage of delignification, was reflected in the high selectivity index (1.27). Slightly lower delignification selectivity was obtained after raspberry fermentation by *P*. *pulmonarius*, while much lower values were noted on the grapevine sawdust (Table 1).

**Table 1:** Extent of pomiculture residue depolymerization by *Pleurotus eryngii* and *P. pulmonarius*

Pomiculture residue	Studied samples	Sample weight (g)	Fibres composition of samples (g)			Dry matter loss (%)	Extent of polymers degradation (%)			Selectivity index
			Lignin	Cellulose	Hemicellulose		Lignin	Cellulose	Hemicellulose	
Grapevine sawdust	Control^1^	6.00	1.36	2.65	0.696	0.00	–	–	–	–
	*P. eryngii*	5.36	1.29	2.13	0.659	10.67^c^	9.46^d^	19.53^b^	25.65^c^	0.48^c^
	*P. pulmonarius*	5.08	1.33	2.12	0.685	15.33^b^	6.44^d^	19.99^b^	22.75^c^	0.32^c^
Plum sawdust	Control^1^	6.00	1.84	2.54	1.37	0.00	–	–	–	–
	*P. eryngii*	5.29	1.54	1.96	0.82	11.83^c^	15.95^c^	23.08^a^	39.69^b^	0.69^b^
	*P. pulmonarius*	5.24	1.48	1.91	0.55	12.67^c^	19.67^b^	24.89^a^	59.44^a^	0.79^b^
Raspberry sawdust	Control^1^	6.00	1.20	2.16	1.31	0.00	–	–	–	–
	*P. eryngii*	5.09	0.96	1.82	0.86	15.17^b^	19.82^b^	15.62^c^	34.61^b^	1.27^a^
	*P. pulmonarius*	4.67	0.92	1.73	0.72	22.17^a^	23.72^a^	19.79^b^	44.66^b^	1.20^a^

The values with the same superscript letter in the same column are not significantly different (*p* < 0.05)

^1^Untreated plant residue

## Discussion

This study provided complete lignocellulolytic profiles of *Pleurotus eryngii* and *P. pulmonarius* during the solid-state fermentation of three common pomiculture lignocellulosic wastes. Despite the quantities produced annually, especially in traditionally fruit-growing regions, as well as the evident disposal problems, grapevine-, plum- and raspberry sawdusts have not yet been studied as substrates for mushroom cultivation and inducers of their lignocellulolytic enzyme cocktails.

The presented results make a significant contribution to the profiling of the ligninolytic potential of well-known white-rot species but on new lignocellulosic substrates, thus clarifying the connection between enzymes production and lignin degradation. Numerous studies have investigated the factors influencing the expression of the ligninolytic enzyme system and have shown that the selection of species/strain, the composition of lignocellulosic material, as well as the type and duration of cultivation significantly affect the activity of ligninolytic enzymes (Richard 1996; de Souza et al. 1999; Arora and Gill 2000; Fenice et al. 2003; Songulashvili et al. 2006). Variations in the substrate type promote the synthesis of various oxidizing enzymes as well as their contribution to complex enzyme cocktails (Elisashvili et al. 2008; Simonić et al. 2010). Numerous studies have already shown the high dependence of the efficiency of the ligninolytic enzyme system of *Pleurotus* spp. on the type of lignocellulosic waste (Adebayo and Martínez-Carrera 2015; Ćilerdžić et al. 2017). Also, Dong et al. (2013) noted extremely high MnP activity (150,000 U L^−1^) in *P. ostreatus* after cultivation on sugar cane substrate, and Xie et al. (2016) after the fermentation of ramie stalks with *P. eryngii* (~ 75,000 U L^−1^), which are far higher than the maximum values obtained in our study. On the other hand, Akpinar and Urek (2014), Inácio et al. (2015), and Wyman et al. (2018) reported similar MnP activities (in the range of 70–570 U L^−1^) synthesized by *P. eryngii*, *P. pulmonarius* and *P. ostreatus* during cultivation on apricot and pomegranate wastes from fruit juice production, as well as orange peels and cornstalks. The influence of plant waste composition and cultivation type on the activity of Mn-oxidizing peroxidases in many white-rot fungi has been the subjects of numerous studies (Stajić et al. 2004; Palma et al. 2016; Sekan et al. 2019). The species of the genus *Pleurotus* tested in our study were incomparably better VP producers than those reported by Stajić et al. (2004) after the submerged fermentation of mandarin peels and solid-state fermentation of grapevine sawdust. On the other hand, significantly higher VP activities were reported by Palma et al. (2016), after the liquid cultivation of *P. eryngii* in a glucose-enriched medium (~ 1800 U L^−1^), and after its solid-state cultivation on banana peels (~ 10,000 U L^−1^). However, *P. eryngii* HAI 1017 synthesized slightly lower active VP isoforms than *P. eryngii* HAI 507 after the solid-state fermentation of wheat straw (Knežević et al. 2013), which at the same time was several-fold higher than those of *P. ostreatus* HAI 592 from the same study. Laccases have been the subject of extensive research for many years, and recently fungal laccases have become particularly interesting due to their exceptional potential for a wide range of applications in numerous biotechnological processes (Paramjeet et al. 2018). Recent research studies are especially focused on the application of genetic engineering to obtain enzymes with the best characteristics, such as higher activity, stability, and yield. However, considerable emphasis is still placed on optimizing the cultivation conditions for the production of highly active forms of laccase in white-rot fungi known to be the best producers of this enzyme. In contrast to the activities of laccases produced during the submerged cultivation of *P. eryngii* and *P. pulmonarius* in a mandarin peel-based medium and their solid cultivation on grapevine sawdust (Stajić et al. 2006), the values obtained in our study were considerably higher. Likewise, glucose and fructose stimulated the synthesis of highly active isoforms of this enzyme in *P. sajor-caju* (37,000 U L^−1^ and 36,000 U L^−1^, respectively) (Bettin et al. 2009), sugar cane residues in *P. ostreatus* (~ 35,000 U L^−1^) (Dong et al. 2013), ramie stalks in *P. eryngii* (~ 15,000 U L^−1^) (Xie et al. 2016) and orange peels in *P. pulmonarius* (~ 10,000 U L^−1^) (Inácio et al. 2015; de Freitas et al. 2017), but to a considerably lower degree than in our study.

A positive correlation between enzymes activities and the extent of depolymerization was not observed in this study, i.e., higher enzyme activity did not mean a higher degree of degradation of the pomiculture residues, which was in line with the results of previous studies (Knežević et al. 2013, 2016; Ćilerdžić et al. 2016b, 2017). This can be explained by the length of cultivation, i.e., by the point at which the activity of some ligninolytic enzymes could significantly decrease. Namely, the onset of enzymes synthesis corresponds to the so-called colonization phase associated with cell wall opening and the initiation of lignin degradation by reactive oxygen species, while enzymatic delignification occurs much later. Due to the lack of clarity regarding the lignocellulose degradation mechanism, today a large number of studies focus on this issue in the aim of creating more efficient processes, i.e., reducing time and energy consumption. An analysis of the genome sequences of ligninolytic enzyme producers showed the absence of a unique set of these enzymes, with composition differing from species to species (Paramjeet et al. 2018). Several genes in the fungal genome encode laccase synthesis, including those that are continuously expressed and those whose transcription depends on environmental conditions and the physiological predisposition of the species (Collins and Dobson 1997; Palmieri et al. 2000; Jiang et al. 2013). Previous studies have also shown the dependence of the number of laccase isoforms produced in *Pleurotus* spp. on the cultivation substrate type (Sannia et al. 1986; Palmieri et al. 1993; Youn et al. 1995; Muñoz et al. 1997a). Thus, the number of isoforms in *P. eryngii* ranged from two, after submerged cultivation in glucose/ammonium tartrate medium and the solid-state fermentation of wheat straw (Muñoz et al. 1997b; Ćilerdžić et al. 2017), to three, following the solid-state fermentation of grapevine sawdust (Stajić et al. 2006), up to four after submerged cultivation in potato/yeast extract medium (Palmieri et al. 1997). Similarly, *P*. *pulmonarius* synthesized only one isoform on wheat straw and two on wheat bran (Marques de Souza and Peralta 2003; Ćilerdžić et al. 2017), and *P. ostreatus* three isoforms after the fermentation of grapevine sawdust (Stajić et al. 2006), while Palmieri et al. (1997) detected as many as four isoforms after cultivation in dextrose/potato extract/yeast extract medium. On the other hand, during cultivation in the same medium, *P. florida* and *P. nebrodensis* produced two and three isoforms, respectively (Das et al. 2001; Yuan et al. 2016).

Previous studies have already shown the potential of species of the genus *Pleurotus* to hydrolyze various lignocellulosic wastes (Zhang et al. 2002; Salmones et al. 2005; Alborés et al. 2006; Goyal and Sony, 2011). Thus, Goyal and Sony (2011) noted the ability of *P. florida*, *P. ostreatus*, and *P. sajor-caju* to synthesize endo- and exo-cellulases as well as β-glucosidases during submerged cultivation in a synthetic medium with carboxymethyl cellulose or wheat straw as carbon sources. However, *P. florida* proved to be the best producer of highly active β-glucosidases, whose activity on the 5^th^ day of cultivation reached the level of 1066 U L^−1^. On the other hand, Ekundayo et al. (2017) showed the high potential of *P. pulmonarius* DBUI002 and *P. ostreatus* DBUI14 to synthesize highly active endo-cellulases (~ 9000 U L^−1^ and 15,000 U L^−1^, respectively), exo-cellulases (~ 9000 U L^−1^ and 13,000 U L^−1^, respectively) and β-glucosidase in particular (~ 32,000 U L^−1^) after 9 days of fermentation of corn cobs and rice bran. However, the *P. pulmonarius* strain used by Inácio et al. (2015) produced far less active endo-cellulases (600 U L^−1^) as well as xylanases (700 U L^−1^) even after 45 days of cultivation on orange peels. Sherief et al. (2010) also detected low β-glucosidase activities in *P. ostreatus* (90 U L^−1^) and *P. sajor-caju* (87 U L^−1^) after the solid-state fermentation of banana peels. The observed differences in the activity of cellulolytic enzymes in *Pleurotus* spp. can be explained by the genetic basis of the species/strain, the type, and composition of the substrate, as well as the fermentation period. Thus, Jørgensen et al. (2007) and Goyal and Sony (2011) explained the decrease or even loss of activity of these enzymes within a certain cultivation period by their inactivation, denaturation, or degradation.

## Conclusions

The results from this study showed, for the first time, that lignocellulosic residues (especially grapevine and raspberry sawdust) can be successfully used as substrates for the cultivation of *P*. *pulmonarius* and *P*. *eryngii*. When treated with *Pleurotus* spp., these commonly available pomiculture residues, have the potential to generate byproducts of nutritional and medicinal value, in addition to the production of lignocellulosomes that can be used in numerous biotechnological processes, such as bioethanol production. The cultivation of *Pleurotus* spp. on lignocellulosics results in the consumption of part of the fermentable sugars, which suggests that additional research is needed to optimize the resulting cocktail for the treatment of the intact lignocellulosic substrate.

## Data Availability

All data generated or analyzed during this study are included in this published article and its additional files.

## Abbreviations

dH_2_O: Distilled water
MnP: Mn-dependent peroxidase
VP: Versatile peroxidase
ABTS: 2,2′-Azino-bis-[3-ethyltiazoline-6-sulfonate]
DNS: Dinitrosalicylic acid reagent
IEF: Isoelectric focusing
NDF: Neutral detergent fibers
ADF: Acidic detergent fibers
LC: Lignin content
pI: Isoelectric point
